# Associations Between Social Determinants of Health and Cardiovascular Health of U.S. Adult Cancer Survivors

**DOI:** 10.1016/j.jaccao.2023.07.010

**Published:** 2023-10-24

**Authors:** Danish Iltaf Satti, Jeffrey Shi Kai Chan, Edward Christopher Dee, Yan Hiu Athena Lee, Abraham Ka Chung Wai, Sourbha S. Dani, Salim S. Virani, Michael D. Shapiro, Garima Sharma, Tong Liu, Gary Tse

**Affiliations:** a Cardio-Oncology Research Unit, Cardiovascular Analytics Group, PowerHealth Limited, Hong Kong SAR; b Division of Cardiology, Johns Hopkins School of Medicine, Baltimore, Maryland, USA; c Department of Radiation Oncology, Memorial Sloan Kettering Cancer Center, New York, New York, USA; d Emergency Medicine Unit, Faculty of Medicine, The University of Hong Kong, Hong Kong SAR; e Division of Cardiovascular Medicine, Comparative Effective Research Institute, Lahey Hospital and Medical Center, Burlington, Massachusetts, USA; f Aga Khan University, Karachi, Pakistan; g Texas Heart Institute, Baylor College of Medicine, Houston, Texas, USA; h Center for Prevention of Cardiovascular Disease, Section on Cardiovascular Medicine, Wake Forest University School of Medicine, Winston-Salem, North Carolina, USA; i Inova Heart and Vascular Institute, Inova Fairfax Medical Campus, Falls Church, Virginia, USA; j Tianjin Key Laboratory of Ionic-Molecular Function of Cardiovascular Disease, Department of Cardiology, Tianjin Institute of Cardiology, Second Hospital of Tianjin Medical University, Tianjin, China; k School of Nursing and Health Studies, Hong Kong Metropolitan University, Hong Kong SAR

**Keywords:** cancer, cardiovascular disease, cardiovascular health, social determinants of health

## Abstract

**Background:**

Relationships between the social determinants of health (SDOH) and cardiovascular health (CVH) of cancer survivors are underexplored.

**Objectives:**

This study sought to investigate associations between the SDOH and CVH of adult cancer survivors.

**Methods:**

Data from the U.S. National Health Interview Survey (2013-2017) were used. Participants reporting a history of cancer were included, excluding those with only nonmelanotic skin cancer, or with missing data for any domain of SDOH or CVH. SDOH was quantified with a 6-domain, 38-item score, consistent with the Centers for Disease Control and Prevention recommendations (higher score indicated worse deprivation). CVH was quantified based on the American Heart Association’s Life’s Essential 8, but due to unavailable detailed dietary data, a 7-item CVH score was used, with a higher score indicating worse CVH. Survey-specific multivariable Poisson regression was used to test associations between SDOH quartiles and CVH.

**Results:**

Altogether, 8,254 subjects were analyzed, representing a population of 10,887,989 persons. Worse SDOH was associated with worse CVH (highest vs lowest quartile: risk ratio 1.30; 95% CI: 1.25-1.35; *P* < 0.001), with a grossly linear relationship between SDOH and CVH scores. Subgroup analysis found significantly stronger associations in younger participants (*P*_interaction_ = 0.026) or women (*P*_interaction_ = 0.001) but without significant interactions with race (*P*_interaction_ = 0.051). Higher scores in all domains of SDOH were independently associated with worse CVH (all *P* < 0.001). Higher SDOH scores were also independently associated with each component of the CVH score (all *P* < 0.05 for highest SDOH quartile).

**Conclusions:**

An unfavorable SDOH profile was independently associated with worse CVH among adult cancer survivors in the United States.

Recent advances in cancer care have led to significantly improved cancer survival rates. As a result, the population of cancer survivors is growing.^1^ In 2022, there were an estimated 18.1 million cancer survivors in the United States (ie, approximately 5% of the population).^2^ Compared with the general population, cancer survivors have increased risks of cardiovascular diseases (CVDs) and cardiovascular mortality, resulting from overlapping risk factors underlying cancer and CVD^3^, ^4^, ^5^, ^6^ and cancer therapy–related cardiotoxicity.^7^, ^8^, ^9^, ^10^ Therefore, cardiovascular care for these patients is increasingly important.

The increased CVD burden among cancer survivors may not be entirely attributable to traditional cardiovascular risk factors.^7^ Studies have highlighted associations of socioeconomic status with CVD risk factors and mortality in the general population.^11^, ^12^, ^13^ Therefore, it is plausible that social determinants of health (SDOH)—encompassing socioeconomic, environmental, and psychosocial factors that influence health—are also associated with CVD in cancer survivors. However, despite efforts to address SDOH-related cardiovascular health (CVH) disparities,^14^ the relationship between SDOH and CVH among cancer survivors remains underexplored. This knowledge gap is particularly relevant as cancer affects both SDOH^15^ and CVH,^16^ meaning that associations between CVH and SDOH observed in other populations may not be directly extrapolated to cancer survivors. Hence, this study aimed to investigate the association between SDOH and CVH among cancer survivors.

## Methods

### Data source

The National Health Interview Survey (NHIS) is an annual household survey conducted by the National Center for Health Statistics/Centers for Disease Control and Prevention, collecting health data for noninstitutionalized civilian adults.^17^ Utilizing multistage probability sampling, the NHIS generates representative estimates for the noninstitutionalized U.S. population.^17^ The NHIS uses sampling weights that account for the complex survey design, including stratification, clustering, and oversampling of certain population groups. These weights are calculated to ensure that the estimates derived from the survey data accurately reflect the characteristics of the noninstitutionalized U.S. population.^18^ Harmonized data were obtained through the Integrated Public Use Microdata Series Health Survey database.^19^ Because all data used were deidentified and publicly available, it was exempt from review by an Institutional Review Board.

### Study population

We analyzed NHIS data from 2013 to 2017 because only these iterations of the NHIS contained all variables required in the ascertainment of CVH score and SDOH score (detailed subsequently). We included adults (≥18 years of age) reporting a diagnosis of cancer, defined as patients who responded “yes” when asked if they had ever been told “by a doctor or other health professional that [they] had cancer or a malignancy of any kind.” Those reporting a diagnosis of nonmelanoma skin cancer were excluded, consistent with other cancer survivorship studies.^20^^,^^21^ Those with missing data for any domain of SDOH or CVH, or any of the prespecified covariates (sex, age, race, sexual orientation, and the presence of any known cardiac condition) were also excluded.

### Ascertainment of CVH

The primary outcome was CVH, quantified by American Heart Association’s Life’s Essential 8 model.^22^ Because the NHIS does not include detailed dietary data, the score comprised 7 binary domains/risk factors (hypertension, diabetes mellitus, hypercholesterolemia, current smoking, physical activity, inappropriate sleep, and obesity). Current smoking status was self-reported. Obesity was defined as body mass index ≥30 kg/m^2^. Insufficient physical activity was defined as not engaging in ≥75 min/wk of vigorous exercise, ≥150 min/wk of moderate intensity exercise or combination, or a total combination of ≥150 min/wk of moderate intensity/vigorous exercise. Inappropriate sleep duration was defined as <6 hours or >10 hours of sleep on average per night. Each of the 7 CVH domains was coded as 0 (absence of a risk factor) or 1 (presence of a risk factor), with a maximum composite CVH score of 7. A higher composite score indicated worse CVH. This score has been published previously.^23^

### Ascertainment of SDOH

We developed a comprehensive SDOH framework based on the 6 domains defined by the Kaiser Family Foundation: economic stability, neighborhood, community and social context, food poverty, education, and access to health care.^24^ Using NHIS data, we identified 38 individual components across these domains (Supplemental Table 1). Each component was classified as favorable or unfavorable, with a value of 0 assigned to the former and 1 to the latter, with a maximum score of 38. To calculate an aggregate SDOH score, we added the scores for individual components. Consequently, a higher aggregate SDOH score indicated worse deprivation. The aggregate SDOH score was used to divide the study population into quartiles, with the first quartile representing the least deprived (lowest SDOH scores) and the fourth quartile representing the most deprived (highest SDOH scores). This score has been previously published.^23^^,^^25^

### Statistical analyses

Survey-specific statistics including sampling weights (divided by the number of survey years included, as per NHIS recommendations) and stratification by the survey year were used to obtain estimates representative of the U.S. population. Continuous variables were described as weighted mean ± weighted SD. Multivariable Poisson regression was used to test the relationships between the SDOH score (in quartiles) and CVH, with the first quartile as reference, and with risk ratio (RR) (“risk” refers to the risk of having a worse CVH score) and the corresponding 95% CI as summary statistics. Regressions were adjusted for prespecified covariates that were part of the self-reported NHIS data: sex, age, race, sexual orientation, and the presence of any known cardiac condition (self-reported history of any heart condition or disease). A 5-knot restricted cubic spline was used to assess the linearity of the association between the SDOH score (as a continuous variable) and the CVH score, with knots placed at the 5th, 27.5th, 50th, 72.5th, and 95th percentiles, as recommended by Harrell.^26^

To further understand whether any of the domains of SDOH had particularly strong associations with CVH and vice versa, multivariable Poisson regression was used to explore these relationships. Individual domains of the SDOH score containing ≥3 subitems were analyzed as both a continuous and a categorical variable. Wherever the data distribution allowed, these variables were analyzed as quartiles. Fewer categories were used wherever meaningful quartiles could not be generated. Multivariable logistic regression (with OR and the corresponding 95% CI as summary statistics) or Poisson regression was used as appropriate.

Four prespecified subgroup analyses were performed to further delineate the relationship between SDOH score (as quartiles) and CVH score, stratifying for age (18-45 years vs 46-64 years vs ≥65 years), sex (male vs female), race (White vs races other than White), and cancer sites (breast, prostate, lung, colon/rectum, and skin [melanoma], which were the 5 most common sites for incident cancer in the United States).^27^

Three prespecified sensitivity analyses were performed. First, although the American Heart Association’s composite CVH score combined different domains with equal weighting, it is unclear whether each domain has equal prognostic values. Thus, each unit increment in CVH score may not be prognostically equal. Therefore, a sensitivity analysis was performed for the CVH score using ordinal logistic regression instead, with OR and the corresponding 95% CI as summary statistics. Second, to further remove and thus clarify the effects that known cardiac conditions may have on the observed associations, a sensitivity analysis was performed in which only patients without any known cardiac condition were analyzed.

The third sensitivity analysis explored the relationship between SDOH (in quartiles) and varying definitions of CVH by using multivariable Poisson regression. As the definition of CVH is still evolving, testing different potential definitions may better reflect the robustness of the observed associations. In this analysis, excessive alcohol use was added as a CVH component, defined as >14 drinks/wk (for men) or >7 drinks/wk (for women) in the past year.^28^

Finally, as worse CVH might have been due to better detection by higher rates of cardiometabolic work-up, a post hoc exploratory analysis was performed to explore the association between the SDOH score (in quartiles) and a self-reported history of having had blood pressure, fasting blood glucose, and cholesterol checked within the past year. Multivariable logistic regression was used for this analysis.

All *P* values were 2-sided, with a *P* value <0.05 considered statistically significant. Because participants with missing values were excluded, the study population had no missing data. All analyses were performed using version 16.1 (StataCorp LLC).

## Results

**Figure 1:**
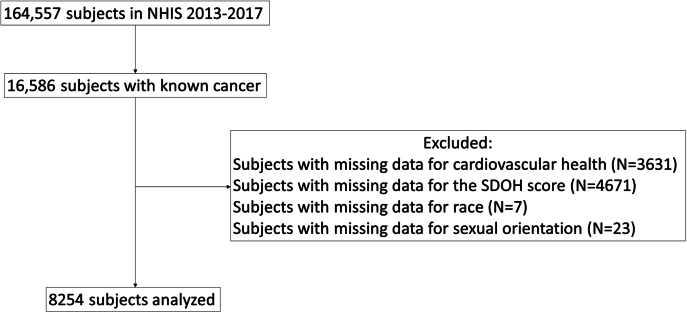
Study Flowchart This summarizes this study’s subject flow. NHIS = National Health Interview Survey; SDOH = social determinants of health.

Of the 16,586 subjects with known cancer in NHIS 2013-2017, 8,254 were analyzed after applying the exclusion criteria (Figure 1), representing a weighted population of 10,887,989 persons. The distribution of the SDOH score is visualized in Supplemental Figure 1, with a weighted mean score of 5.3 ± 4.2. Subjects in the first quartile of the SDOH score had a score of 0 to 2, the second quartile had a score of 3 to 4, the third quartile had a score of 5 to 7, and the fourth quartile had a score of 8 to 28. The per component distribution of the SDOH score is detailed in Supplemental Table 2. The distribution of the CVH score is visualized in Supplemental Figure 2, with a weighted mean score of 2.9 ± 1.5. Characteristics of the included subjects are summarized in Table 1. Characteristics of subjects were also tabulated against the included subjects in Supplemental Tables 3 and 4. The included and excluded subjects were generally comparable, except that the included subjects were older, were more commonly male, had higher rates of hypercholesterolemia, and less commonly had low family income compared with the excluded subjects.

**Table 1:** Demographics and Components of Cardiovascular Health in the Included Subjects

	Overall (N = 8,254)	SDOH			
		Quartile 1 (n = 2,493)	Quartile 2 (n = 2,010)	Quartile 3 (n = 1,801)	Quartile 4 (n = 1,950)
Weighted sample size	10,887,989	3,233,506	2,619,067	2,433,199	2,602,216
Demographics					
Age					
18-45 y	581 (7.0)	53 (2.1)	76 (3.8)	156 (8.7)	296 (15.2)
46-64 y	2,524 (30.6)	478 (19.2)	508 (25.3)	607 (3.7)	931 (47.7)
65 y or above	5,149 (62.4)	1,962 (78.7)	1,426 (71.0)	1,038 (57.6)	723 (37.1)
Male	3,755 (45.5)	1,343 (53.9)	934 (46.5)	772 (42.9)	706 (36.2)
Race					
American Indian/Alaskan native	39 (0.5)	8 (0.3)	3 (0.2)	13 (0.7)	15 (0.8)
Black/African American	526 (6.4)	122 (4.9)	101 (5.0)	117 (6.5)	186 (9.5)
Asian	154 (1.9)	41 (1.6)	34 (1.7)	40 (2.2)	39 (2.0)
White	7,405 (89.7)	2,299 (92.2)	1,842 (91.6)	1,604 (89.1)	1,660 (85.1)
Multiple race	130 (1.6)	23 (0.9)	30 (1.5)	27 (1.5)	50 (2.6)
Heterosexual	7,995 (96.9)	42 (1.7)	55 (2.7)	54 (3.0)	108 (5.5)
Type of cancer (not mutually exclusive)					
Breast	1,502 (18.2)	438 (17.6)	364 (18.1)	337 (18.7)	365 (18.7)
Prostate	1,094 (13.3)	442 (17.7)	287 (14.3)	210 (11.7)	155 (8.0)
Lung	274 (3.3)	78 (3.1)	59 (2.9)	61 (3.4)	76 (3.9)
Colorectal	531 (6.4)	166 (6.7)	122 (6.1)	107 (5.9)	136 (7.0)
Skin (melanoma)	646 (7.8)	219 (8.8)	164 (8.2)	136 (7.6)	127 (6.5)
Other types	2,687 (32.6)	622 (25.0)	588 (29.3)	628 (64.9)	849 (43.5)
Unknown	2,097 (25.4)	718 (28.0)	553 (27.5)	448 (24.9)	378 (19.4)
CVH domains					
Hypertension	4,883 (59.2)	1,530 (61.4)	1,182 (58.8)	1,065 (59.1)	1,106 (56.7)
Diabetes mellitus	1,955 (23.7)	502 (20.1)	474 (23.6)	447 (24.8)	532 (27.3)
Hypercholesterolemia	4,411 (53.4)	1,394 (55.9)	1,099 (54.7)	944 (52.4)	974 (50.0)
Smoking	4,313 (52.3)	1,236 (49.6)	1,017 (50.6)	943 (52.4)	1,117 (27.3)
Physical inactivity	5,204 (63.1)	1,498 (60.1)	1,188 (59.1)	1,153 (64.0)	1,365 (70.0)
Inadequate sleep	1,239 (15.0)	236 (9.5)	252 (12.5)	278 (15.4)	473 (24.3)
Obesity	2,702 (32.7)	687 (27.6)	577 (28.7)	636 (35.3)	802 (41.1)
Excessive alcohol use	458 (5.6)	126 (7.9)	117 (9.0)	97 (8.9)	118 (10.0)

Values are n or n (%). Percentages are unweighted.

CVH = cardiovascular health; SDOH = social determinants of health.

### Association between SDOH and CVH

**Figure 2:**
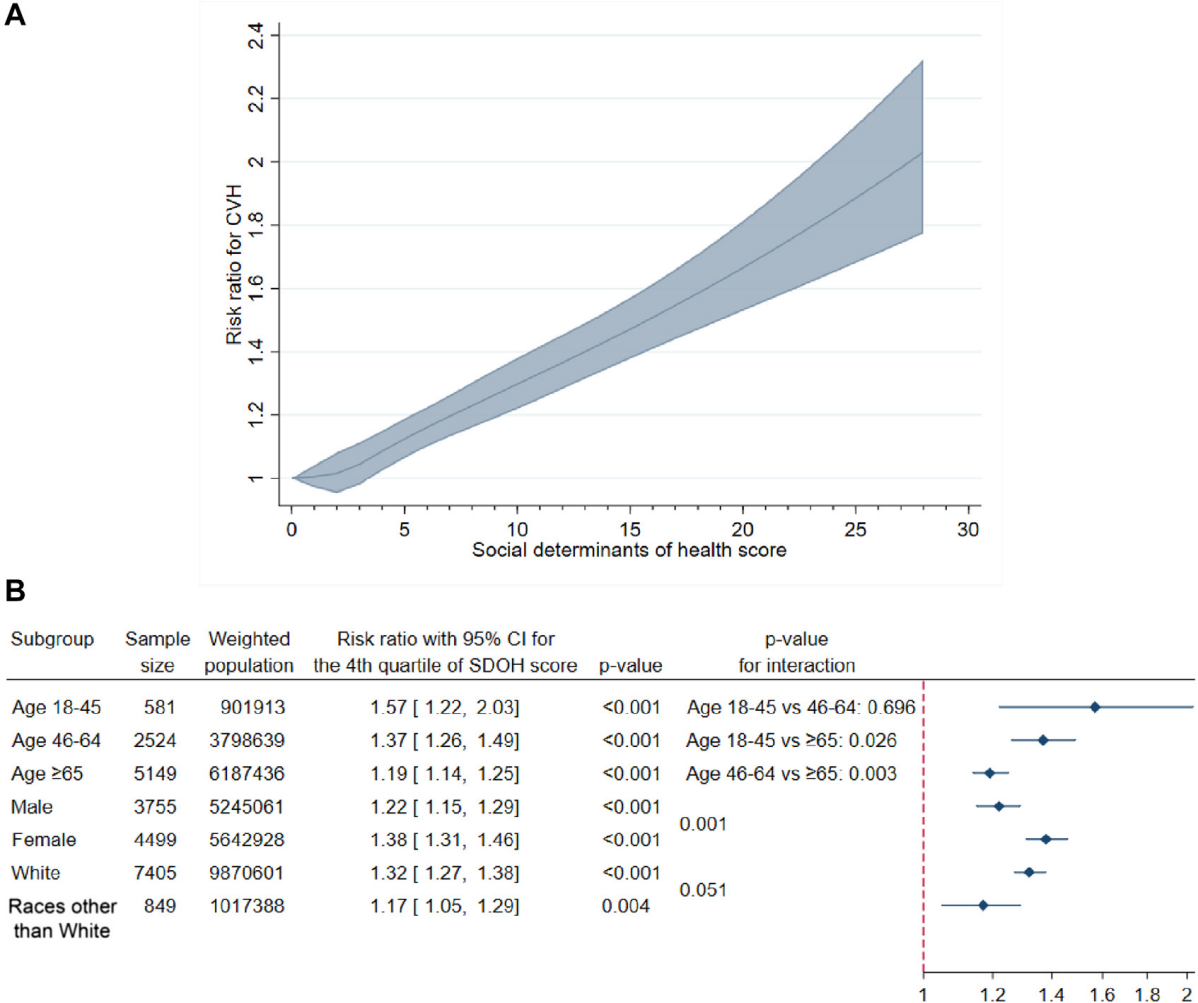
Summary of Key Results (A) A 5-knot restricted cubic spline shows a direct and grossly linear relationship between the social determinants of health (SDOH) score and the primary outcome (cardiovascular health [CVH]). (B) Subgroup analyses for the primary outcome show particularly strong SDOH-CVH associations in younger or female cancer survivors. Adjusted risk ratios and 95% CIs from multivariable Poisson regression are displayed.

The highest (fourth) quartile of the SDOH score was independently associated with a higher risk of having worse CVH (RR: 1.30; 95% CI: 1.25-1.35; *P* < 0.001) (Table 2). The relationship between the SDOH score and CVH (Figure 2A) was grossly linear.

**Table 2:** Associations Between the SDOH Score and CVH Score

SDOH Score	Primary Outcome (CVH Score)^a^
Quartile 1	1.00 (reference)
Quartile 2	1.04 (1.00-1.08), 0.085
Quartile 3	1.13 (1.09-1.18), <0.001
Quartile 4	1.30 (1.25-1.35), <0.001

Values are adjusted risk ratio (95% CI), *P* value.

Abbreviations as in Table 1.

a Adjusted for sex, age, race, sexual orientation, and the presence of any known cardiac condition.

**Figure 3:**
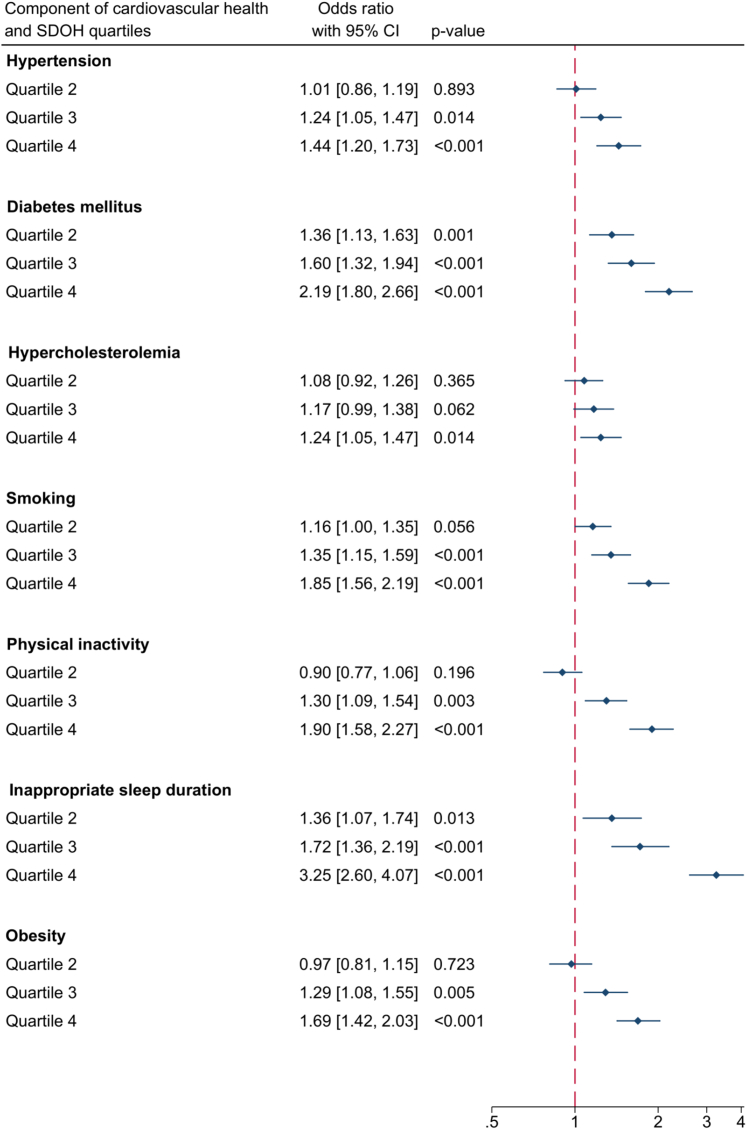
Associations for the Components of CVH The forest plot shows significant associations between the SDOH score and all components of the CVH score. Adjusted ORs and 95% CIs from multivariable logistic regression are displayed. Abbreviations as in Figure 2.

Except for the education domain, higher values in all other domains of the SDOH score were independently associated with worse CVH (Table 3), with the strongest association observed for food insecurity (RR: 1.38; 95% CI: 1.31-1.45; *P* < 0.001). Meanwhile, independent associations were noted between the highest quartile of the SDOH score and all components of CVH (Figure 3), with the strongest association observed for inappropriate sleep duration (OR: 3.25; 95% CI: 2.60-4.07; *P* < 0.001).

**Table 3:** Associations Between Individual Domains of the SDOH Score and CVH

Domain of the SDOH Score	Number of Domains	Primary Outcome (CVH Score)^a^	
		Adjusted Risk Ratio (95% CI)	*P* Value
Economic stability	13		
As a continuous variable		1.04 (1.03-1.04)	<0.001
As quartiles			
Quartile 1		1.00 (reference)	
Quartile 2		1.03 (0.99-1.07)	0.14
Quartile 3		1.11 (1.06-1.15)	<0.001
Quartile 4		1.28 (1.23-1.33)	<0.001
Neighborhood, physical environment, and social cohesion	5		
As a continuous variable		1.06 (1.05-1.07)	<0.001
As tertiles^b^			
Tertile 1		1.00 (reference)	
Tertile 2		1.06 (1.03-1.10)	0.001
Tertile 3		1.20 (1.16-1.24)	<0.001
Community and social context	1	1.29 (1.20-1.37)	<0.001
Food	1	1.38 (1.31-1.45)	<0.001
Education	7		
As a continuous variable		0.99 (0.98-1.01)	0.48
≤1 domain^b^		1.00 (reference)	
≥2 domains^b^		0.99 (0.96-1.02)	0.39
Health care system	11		
As a continuous variable		1.05 (1.03-1.06)	<0.001
No domain^b^		1.00 (reference)	
Any domain^b^		1.07 (1.03-1.11)	<0.001

RR = risk ratio; other abbreviations as in Table 1.

a Adjusted for sex, age, race, sexual orientation, and the presence of any known cardiac condition.

b Data distribution did not allow meaningful quartiles to be generated.

### Subgroup analyses

After stratifying for age, sex, and race, the SDOH score remained independently associated with CVH (Figure 2B, Supplemental Table 5). The associations were significantly stronger in participants who were younger (18-45 years of age vs ≥65 years of age: *P*_interaction_ = 0.026; 46-64 years of age vs ≥65 years of age: *P*_interaction_ = 0.003). No statistically significant interaction between the SDOH score and race was observed (*P*_interaction_ = 0.051).

Stratification for cancer sites showed largely consistent results, with significant associations for CVH observed for all 5 specified cancer sites (Supplemental Table 5).

### Sensitivity analyses

Results from sensitivity analyses were consistent with the main analyses (Supplemental Table 6). Ordinal logistic regression demonstrated a robust and independent relationship between the highest quartile of the SDOH score and the higher CVH score (OR 2.57; 95% CI: 2.21-2.98; *P* < 0.001). Meanwhile, a similar association was observed for CVH among those without any known cardiac condition (RR: 1.34; 95% CI: 1.27-1.40; *P* < 0.001). Adding excessive alcohol use to CVH as a component of CVH (RR: 1.33; 95% CI: 1.27-1.40; *P* < 0.001) also resulted in consistently strong associations with the SDOH score.

### Exploratory analyses

Post hoc exploratory analyses (Supplemental Table 7) found no significant association between the SDOH score and self-reported history of having had blood pressure (OR for the highest quartile of SDOH: 0.89; 95% CI: 0.50-1.58; *P* = 0.70), fasting blood glucose (OR for the highest quartile of SDOH: 1.07; 95% CI: 0.89-1.28; *P* = 0.47), or blood cholesterol (OR for the highest quartile of SDOH: 0.94; 95% CI: 0.70-1.26; *P* = 0.69) checked within the past year.

## Discussion

Using nationally representative U.S. data, we demonstrated a strong and robust relationship between disadvantaged SDOH profile and suboptimal CVH among cancer survivors, which was particularly prominent among women and younger participants (Central Illustration). To our knowledge, this is the first study to investigate this association among cancer survivors directly.

**Central Illustration:**
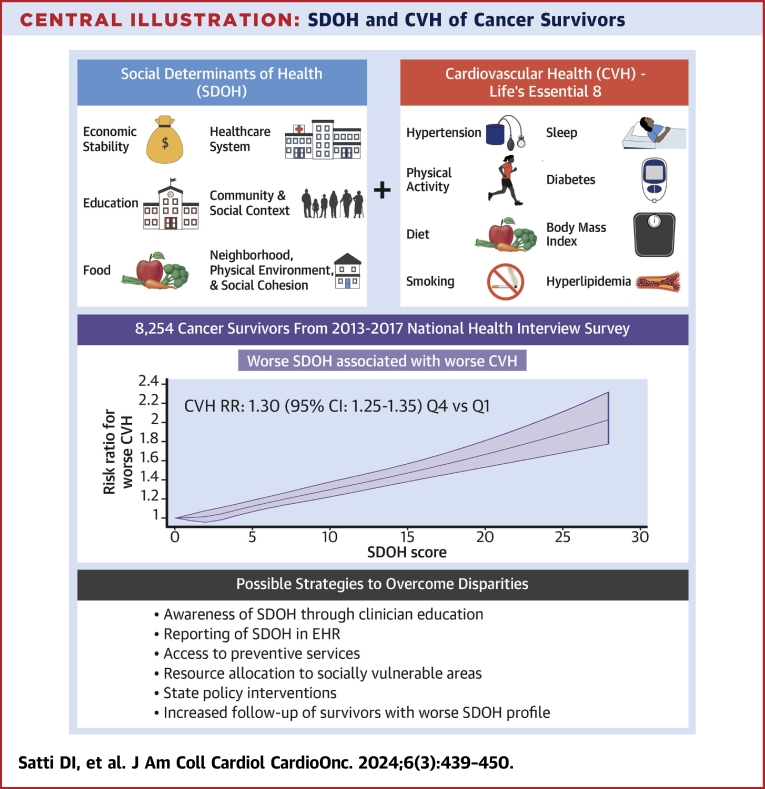
SDOH and CVH of Cancer Survivors The Central Illustration highlights the significant associations observed between various social determinants of health (SDOH) factors and the cardiovascular health (CVH) of cancer survivors. It provides a visual summary of the key findings, emphasizing the impact of SDOH on CVH outcomes. The figure underscores the importance of considering SDOH factors in promoting cardiovascular well-being among cancer survivors and highlights potential areas for targeted interventions and support. EHR = electronic health record; RR = risk ratio.

### Comparison with previous literature

Unfavorable SDOH profiles have been associated with higher prevalence of CVD and cancer-related mortality in the general population.^12^^,^^23^^,^^29^^,^^30^ Previous studies demonstrated that various domains of SDOH influence CVH in complex and variable ways. For example, Makhlouf et al^31^ found that neighborhood walkability and the green space availability were associated with better CVH in the United States. Similarly, in another cross-sectional survey, food insecurity was associated with suboptimal CVH.^32^ Housing insecurity has also been identified to be associated with CVH in the general population.^33^

Although all these SDOH domains are important, only specific facets of SDOH have been investigated for their relationships with CVH in cancer survivors. For instance, Batra et al^34^ found that rural residence, low income, and low education were associated with higher risks of developing CVD in cancer survivors. These were confirmed by Berkman et al,^35^ who found that an annual household income <$50,000 USD was associated with increased odds of CVD in young adult cancer survivors, and Appiah et al,^36^ who showed that breast and gynecologic cancer survivors residing in rural areas had higher risks of cardiovascular mortality.

However, the impact of SDOH extends beyond these few specific factors, and studying individual domains of SDOH in isolation is inadequate because they are likely associated with CVH in complex and intersectional ways. Instead, an aggregate SDOH risk score may better identify and improve care for socially disadvantaged individuals.^37^ Hence, we used a well-established and published aggregate SDOH score, ensuring reliability, robustness, and objectivity. Similar issues may exist for CVH quantification, which is evolving. Therefore, we referenced the American Heart Association's Life's Essential 8 model, which is an evidence-based framework created in 2022 to define and quantify CVH.^22^ Although the original model involved more detailed measurements and included diet, the CVH score used in this study had been previously published and shown to be a robust measurement of CVH.^23^^,^^25^ The use of this CVH score thus ensured robustness of our findings. This was further reinforced by sensitivity analyses, in one of which excessive alcohol use was added to the CVH score in recognition of CVH as an evolving concept.

Importantly, we found that the association between SDOH and CVH was particularly strong among women or young individuals, congruent with previous research on associations between social vulnerability and mortality due to comorbid cancer and CVD.^38^ The worse CVH associated with disadvantaged SDOH may have contributed to such observations for mortality, further emphasizing the need to prioritize interventions that address social and economic disadvantage in female and young cancer survivors.

### Underlying mechanisms

The association between SDOH and CVH is likely multifactorial. However, our exploratory analyses suggested that differences in rates of cardiometabolic work-up within the past year were unlikely to be the driving factor behind this association. We speculate that the adverse association between socioeconomic disadvantage and mental health may be one of the potential mediators.^39^^,^^40^ We previously showed that psychological distress is associated with worse CVH in adult cancer survivors.^41^ Others have also hypothesized that psychological factors mediate associations between social/physical environments and CVD,^42^^,^^43^ which may be positive (eg, social support improving health behaviors among racial/ethnic minority groups by reducing depressive symptoms)^44^^,^^45^ or negative (eg, poorly built environments increasing the risk of CVD via an increased likelihood of mental disorders causing chronic life stress).^46^^,^^47^ Nevertheless, other mediating mechanisms also likely exist. Further studies are required to delineate the drivers of our observations.

### Clinical and policy implications

Our findings have substantial clinical and public health implications, as they underscore the pivotal roles of social, economic, and environmental conditions in determining the CVH of cancer survivors, particularly younger individuals, and women. Our results demonstrated substantial social disparity in CVH among cancer survivors, highlighting the need for comprehensive interventions at various levels to minimize social disparity and ultimately optimize cardiovascular outcomes in this population.

At the clinical level, clinicians must be educated about the strong links between SDOH and CVH in cancer survivors so that those with poor SDOH profiles can be flagged and targeted for specific interventions (Central Illustration). This process may also benefit from better reporting of patients' SDOH within electronic health records, facilitating SDOH profiling and risk stratification, and enabling more in-depth research into this area.

At the health care system and policy levels, investments in health care infrastructure may need to be increased in socially vulnerable areas to ensure equitable access to quality health care. State policies may also need to be recalibrated to enhance cancer survivors’ access to preventive medicine services, such as ensuring continued follow-up care for those with disadvantaged SDOH profiles. Given the interconnected nature of these factors and their cascading downstream effects on health outcomes, national efforts are needed to reduce social disparities in CVH among cancer survivors. This can be achieved within broader programs such as the Centers for Medicare and Medicaid Services initiative,^48^ which focuses on addressing SDOH for Medicare and Medicaid beneficiaries. The Centers for Medicare and Medicaid Services initiative aims to support health care providers in identifying and addressing SDOH factors by screening patients for SDOH risks, providing referrals to community resources, and integrating SDOH interventions into care plans. It also aims to standardize data collection and analysis related to SDOH, as well as collaborations with community organizations and other stakeholders to address SDOH more broadly. Similar programs are needed to target cancer survivors and optimize their CVH.

### Strengths and limitations

We utilized a nationally representative U.S. database, ensuring the generalizability of our findings within the United States, with potential generalizability to other developed countries. In addition, the robustness of our analyses was reinforced by multiple sensitivity analyses, which consistently yielded similar observations. Finally, the use of well-established and published measurement tools of SDOH and CVH ensured objectivity and reliability.

However, our study is not devoid of limitations. First, because all NHIS data are self-reported, they are subject to misreporting, underreporting, and recall bias. Specifically, any history of cancer diagnosis and all components of CVH were self-reported without cross-checking with physicians or against medical records. However, participants who reported a history of cancer were subsequently asked for the cancer type, thereby partly mitigating the risk of misreporting.

Second, as detailed dietary data are available in the NHIS, we were unable to include dietary variables in our assessment of Life's Essential 8. This limitation was partially mitigated in our sensitivity analyses by using excessive alcohol use as a surrogate for poor dietary habits within the CVH component, yielding consistent results.

Third, the cross-sectional design of NHIS precludes any establishment of causality. Although existing evidence predominantly supports the role of SDOH as a predictor of CVH (with the posited direction of the association being from unfavorable SDOH to worse CVH and not otherwise),^49^ it remains possible that worse CVH leads to more unfavorable SDOH via increased medical expenditure, reduced exercise/socialization, or other mechanisms. Future research should explore the potential and implications of reverse causation in the SDOH-CVH-cancer context, as well as address the possibility of residual confounding.

Additionally, although we explored differences in the association between SDOH and CVH between races/ethnicities, very few of the analyzed cancer survivors were races other than White. This was not unexpected because NHIS is representative of the U.S. population, which is predominantly White. The small sample sizes limited the statistical power of the interaction analysis and barred further stratification of races other than White cancer survivors into detailed races/ethnicities. Therefore, with substantially different point estimates, minimally overlapping 95% CIs, and borderline statistical significance, interracial differences in the association between SDOH and CVH could not be definitively excluded. Overall, the demographics of the sampled individuals in NHIS meant that our findings might not be directly generalizable to countries with substantially different racial/ethnic compositions. Larger international studies are needed to corroborate our findings across cancer survivors of different races/ethnicities.

Finally, we acknowledge that the subjects included in our analysis differed in certain aspects from those who were excluded, which may impact generalizability and representativeness of our findings. Large prospective studies with minimal data missingness remain required to verify our findings.

## Conclusions

Among cancer survivors in the United States, an unfavorable SDOH profile was independently associated with worse CVH, especially in young and female subjects. This highlights the need for a comprehensive approach to health care for cancer survivors that considers the broader socioeconomic and environmental factors associated with their CVH.

Perspectives **COMPETENCY IN MEDICAL KNOWLEDGE:** In this 2013-2017 cross-sectional study, unfavorable SDOH profiles were associated with worse CVH in adult cancer survivors. This association was stronger in younger individuals and women.

**TRANSLATIONAL OUTLOOK:** This study highlights the importance of social, economic, and environmental factors that may impact the CVH of cancer survivors. A holistic approach addressing these determinants is needed to improve the CVH needs of this vulnerable population.

## Funding Support and Author Disclosures

This work was partly supported by the Tianjin Key Medical Discipline (Specialty) Construction Project (Project number: TJYXZDXK-029A) and by a grant from the Hong Kong Metropolitan University (Project Reference No. RIF/2022/2.2). The funder played no role in any part of this study. Dr Dee is funded in part through the Cancer Center Support Grant from the National Cancer Institute (P30 CA008748). Dr Sharma is supported by the Blumenthal Scholarship in Preventive Cardiology at the Ciccarone Center for the Prevention of Cardiovascular Disease at Johns Hopkins University School of Medicine and American Heart Association grant 979462. Dr Virani has received grant support from the Department of Veterans Affairs, the World Heart Federation, and the Tahir and Jooma Family; and honoraria from the American College of Cardiology (Associate Editor for *Innovations*). Dr Shapiro has served on scientific advisory boards for Amgen, Ionis, Novartis, and Precision BioScience; and as a consultant for Ionis, Novartis, Regeneron, EmendoBio, and Aidoc. All other authors have reported that they have no relationships relevant to the contents of this paper to disclose.
