# Supplementary material for: Associations Between Social Determinants of Health and Cardiovascular Health of U.S. Adult Cancer Survivors
Source: JACC CardioOncol. 2023 Oct 24;6(3):439–50. doi: 10.1016/j.jaccao.2023.07.010 (PMC11229543; doi:10.1016/j.jaccao.2023.07.010)

**Supplementary Material**

**Associations between Social Determinants of Health and Cardiovascular Health of US Adult Cancer Survivors**

**Table of Contents**

| **Title** | **Description** | **Page(s)** |
| --- | --- | --- |
| Components of the social determinants of health score. | Supplementary Table 1 | 2-8 |
| Unweighted proportion of the included subjects having each component and sub-item of the social determinants of health (SDOH) score. | Supplementary Table 2 | 9 |
| Demographics and components of cardiovascular health in included and excluded subjects. | Supplementary Table 3 | 10-11 |
| Proportions of the included and excluded subjects having each component and sub-item of the social determinants of health (SDOH) score. | Supplementary Table 4 | 12-13 |
| Subgroup analyses for the primary outcome (Cardiovascular Health score). | Supplementary Table 5 | 14 |
| Results of sensitivity analyses. | Supplementary Table 6 | 15 |
| Results of the exploratory analyses exploring associations between the social determinants of health (SDOH) score and self-reported history of cardiometabolic workup within the past year. | Supplementary Table 7 | 16 |
| Weighted distribution of the social determinants of health score. | Supplementary Figure 1 | 17 |
| Weighted distribution of the primary outcome (CVH score). | Supplementary Figure 2 | 18 |

**Supplementary Table 1.** Components of the social determinants of health score.

| **Shorter version of survey items** | **Longer version of survey items** | **Survey Responses** | **Analytic recode** |
| --- | --- | --- | --- |
| **ECONOMIC STABILITY** | | | |
| Employment | What was your employment status as of last week? | Working for pay at a job or business; With a job or business but not at work; Looking for work; Working, but not for pay, at a family-owned job or business; Not working at a job or business and not looking for work | 0 = "Employed or Retired"; 1 = "Never or Previously Employed" |
| Sick Leave | Paid sick leave at current job or most current job | Yes; No | 0 = "Yes"; 1 = "No" |
| Family Income | Ratio of family income to poverty threshold |  | 0 = "Middle/High-income" (≥ 200% of poverty threshold); 1 = "Low-income" (< 200% of poverty threshold) |
| Any Difficulty Paying Medical Bills | In the past 12 months did you/anyone in the family have problems paying or were unable to pay any medical bills? Include bills for doctors, dentists, hospitals, therapists, medication, equipment, nursing home or home care. | Yes; No | 0 = "No"; 1 = "Yes" |
| Unable to Pay Medical Bills | If previous question = Yes: Do you/Does anyone in your family currently have any medical bills that you are unable to pay at all? | Yes; No | 0 = "No"; 1 = "Yes" |
| Cost-related medication non-adherence (positive if any of the following 3 questions' answer was Yes): | | | |
| … Skipped medication doses to save money | During the past 12 months, were any of the following true for you?  …You skipped medication doses to save money | Yes; No | 0 = "No"; 1 = "Yes" |
| … Took less medicine to save money | During the past 12 months, were any of the following true for you?  …you took less medicine to save money | Yes; No | 0 = "No"; 1 = "Yes" |
| … Delayed filling prescription to save money | During the past 12 months, were any of the following true for you?  …You delayed filling a prescription to save money | Yes; No | 0 = "No"; 1 = "Yes" |
| Delayed/Foregone Care due to Cost (positive if any of the following 2 questions' answer was Yes): | | | |
| Delayed Care due to Cost | During the past 12 months, has medical care been delayed for because of worry about the cost? (Do not include dental care) | Yes; No | 0 = "No"; 1 = "Yes" |
| Foregone Care due to Cost | During the past 12 months, was there any time when needed medical care, but did not get it because couldn't afford it? | Yes; No | 0 = "No"; 1 = "Yes" |
| High Financial Distress Composite Score (aggregate score from the following 6 questions);  Worried about … | | | From the aggregate sum of the following 6 items, divided into quartiles:  0 = quartiles 1-3; 1 =  quartile 4 |
| … Money for retirement | How worried are you right now about not having enough money for retirement? | Very worried; Moderately worried; Not too worried; Not worried at all | 0 = "Not too worried/Not worried at all"; 1 = "Mod/Very worried" |
| … Medical costs of illness/accident | How worried are you right now about not being able to pay medical costs of a serious illness or accident? | Very worried; Moderately worried; Not too worried; Not worried at all | 0 = "Not too worried/Not worried at all"; 1 = "Mod/Very worried" |
| … Maintaining standard of living | How worried are you right now about not being able to maintain the standard of living you enjoy? | Very worried; Moderately worried; Not too worried; Not worried at all | 0 = "Not too worried/Not worried at all"; 1 = "Mod/Very worried" |
| … Medical costs of healthcare | How worried are you right now about not being able to pay medical costs for normal healthcare? | Very worried; Moderately worried; Not too worried; Not worried at all | 0 = "Not too worried/Not worried at all"; 1 = "Mod/Very worried" |
| … Paying monthly bills | How worried are you right now about not having enough to pay your normal monthly bills? | Very worried; Moderately worried; Not too worried; Not worried at all | 0 = "Not too worried/Not worried at all"; 1 = "Mod/Very worried" |
| … Paying rent/mortgage/housing costs | How worried are you right now about not being able to pay your rent, mortgage, or other housing costs? | Very worried; Moderately worried; Not too worried; Not worried at all | 0 = "Not too worried/Not worried at all"; 1 = "Mod/Very worried" |
| **NEIGHBOURHOOD, PHYSICAL ENVIRONMENT AND SOCIAL CONTEXT** | | | |
| House Tenure | Is this house/apartment owned or being bought, rented, or occupied by some other arrangement by [you/or someone in your family]? | Owned or being bought; Rented; Other arrangement | 0 = "Own or being bought"; 1 = "Rent/Other arrangement" |
| Neighborhood Quality (Help) | How much do you agree or disagree with the following statements about your neighborhood? Would you say… People in this neighborhood help each other out. | Definitely agree; Somewhat agree; Somewhat disagree; Definitely disagree | 0 = "Agree (Somewhat/Definitely)"; 1 = "Disagree (Somewhat/Definitely)" |
| Neighborhood Quality (Trust) | How much do you agree or disagree with the following statements about your neighborhood? Would you say… People in this neighborhood can be trusted. | Definitely agree; Somewhat agree; Somewhat disagree; Definitely disagree | 0 = "Agree (Somewhat/Definitely)"; 1 = "Disagree (Somewhat/Definitely)" |
| Neighborhood Quality (Close Knit) | How much do you agree or disagree with the following statements about your neighborhood? Would you say… This is a close-knit neighborhood. | Definitely agree; Somewhat agree; Somewhat disagree; Definitely disagree | 0 = "Agree (Somewhat/Definitely)"; 1 = "Disagree (Somewhat/Definitely)" |
| Neighborhood Quality (Accountability) | How much do you agree or disagree with the following statements about your neighborhood? Would you say… There are people I can count on in this neighborhood. | Definitely agree; Somewhat agree; Somewhat disagree; Definitely disagree | 0 = "Agree (Somewhat/Definitely)"; 1 = "Disagree (Somewhat/Definitely)" |
| **COMMUNITY AND SOCIAL CONTEXT** | | | |
| Kessler K6 Scale for High Psychological Distress (derived from the following 6 questions): | | | From the aggregate sum of the following 6 items: 0 = "No psychological distress" (sum ≤ 12); 1 = "Psychological distress" (sum ≥ 13) |
| … Feeling sad | During the past 30 days, how often did you feel …so sad that nothing could cheer you up? | All of the time; Most of the time; Some of the time; A little of the time; None of the time | 0 = "None of the time"; 1  = "A little of the time"; 2  = "Some of the time"; 3 = "Most of the time"; 4 = "All of the time" |
| … Nervous | During the past 30 days, how often did you feel … nervous? | All of the time; Most of the time; Some of the time; A little of the time; None of the time | 0 = "None of the time"; 1  = "A little of the time"; 2  = "Some of the time"; 3 = "Most of the time"; 4 = "All of the time" |
| … Restless/fidgety | During the past 30 days, how often did you feel … restless or fidgety? | All of the time; Most of the time; Some of the time; A little of the time; None of the time | 0 = "None of the time"; 1  = "A little of the time"; 2  = "Some of the time"; 3 = "Most of the time"; 4 = "All of the time" |
| … Restless/fidgety | During the past 30 days, how often did you feel … restless or fidgety? | All of the time; Most of the time; Some of the time; A little of the time; None of the time | 0 = "None of the time"; 1  = "A little of the time"; 2  = "Some of the time"; 3 = "Most of the time"; 4 = "All of the time" |
| … Hopeless | During the past 30 days, how often did you feel … hopeless? | All of the time; Most of the time; Some of the time; A little of the time; None of the time | 0 = "None of the time"; 1  = "A little of the time"; 2  = "Some of the time"; 3 = "Most of the time"; 4 = "All of the time" |
| … Everything was an effort | During the past 30 days, how often did you feel … that everything was an effort? | All of the time; Most of the time; Some of the time; A little of the time; None of the time | 0 = "None of the time"; 1  = "A little of the time"; 2  = "Some of the time"; 3 = "Most of the time"; 4 = "All of the time" |
| … Worthless | During the past 30 days, how often did you feel … worthless? | All of the time; Most of the time; Some of the time; A little of the time; None of the time | 0 = "None of the time"; 1  = "A little of the time"; 2  = "Some of the time"; 3 = "Most of the time"; 4 = "All of the time" |
| **FOOD** | | | |
| Food Insecurity (based on US Dept. of Agriculture Standardized Questionnaire) | | | From the aggregate sum of the following 10 items:  0 = "Food Secure" (sum ≤ 2); 1 = "Food Insecure" (sum ≥ 3) |
| … Worried food would run out before got money to buy more | [fill 2: I/We] worried whether [fill 3: my/our] food would run out before [fill 4: I/we] got money to buy more. Was that often true, sometimes true, or never true for [fill 1: you/your family] in the last 30 days? | Often true; Sometimes true; Never true | 0 = "Never true"; 1 = "Sometimes true/Often true" |
| … Food did not last before had money to get more | The food that [fill 1: I/we] bought just didn't last, and [fill 1: I/we] didn't have money to get more. Was that often true, sometimes true, or never true for [fill 2: you/your family] in the last 30 days? | Often true; Sometimes true; Never true | 0 = "Never true"; 1 = "Sometimes true/Often true" |
| … Could not afford to eat balanced meals | [fill 1: I/We] couldn't afford to eat balanced meals. Was that often true, sometimes true, or never true for [fill 2: you/your family] in the last 30 days? | Often true; Sometimes true; Never true | 0 = "Never true"; 1 = "Sometimes true/Often true" |
| … Cut size or skipped meals because not enough money | In the last 30 days, did [fill 1: you/you or other adults in your family] ever cut the size of your meals or skip meals because there wasn't enough money for food? | Yes; No | 0 = "No"; 1 = "Yes" |
| … If above question = Yes: How many days in past month? | In the last 30 days, how many days did this happen? | 01-30 days (continuous response) | 0 = if < 3 days; 1 = if ≥ 3 days |
| … Eat less than felt should because not enough money | In the last 30 days, did you ever eat less than you felt you should because there wasn't enough money for food? | Yes; No | 0 = "No"; 1 = "Yes" |
| … Hungry but did not eat because not enough money | In the last 30 days, were you ever hungry but didn't eat because there wasn't enough money for food? | Yes; No | 0 = "No"; 1 = "Yes" |
| … Lose weight because not enough money for food | In the last 30 days, did you lose weight because there wasn't enough money for food? | Yes; No | 0 = "No"; 1 = "Yes" |
| … Not eat for a whole day because not enough money for food | In the last 30 days, did [fill 1: you/you or other adults in your family] ever not eat for a whole day because there wasn't enough money for food? | Yes; No | 0 = "No"; 1 = "Yes" |
| … If above question = Yes: How many days in past month? | In the last 30 days, how many days did this happen? | 01-30 days (continuous response) | 0 = if < 3 days; 1 = if ≥ 3 days |
| **EDUCATION** | | | |
| English Language | How well do you speak English? | Very well; Well; Not well; Not at all | 0 = "Well/Very Well"; 1 = "Not well/Not at all" |
| Education Attainment | What is the HIGHEST level of school completed or the highest degree received? | Never attended/kindergarten only; 1st grade; 2nd grade; 3rd grade; 4th grade; 5th grade; 6th grade; 7th grade; 8th grade; 9th grade; 10th grade; 11th grade; 12th grade; GED or equivalent; High school graduate; Some college, no degree; Associate degree: occupational, technical, or vocational program; Associate degree: academic program; Bachelor's degree; Master's degree; Professional school degree; Doctoral degree | 0 = "≥ Some college"; 1 = "≤ High School" |
| Health Information Technology use: Looked up health info on internet | DURING THE PAST 12 MONTHS, have you ever used computers for any of the following …Look up health information on the Internet | Yes; No | 0 = "No"; 1 = "Yes" |
| Health Information Technology use: Filled a prescription online | DURING THE PAST 12 MONTHS, have you ever used computers for any of the following …Fill a prescription | Yes; No | 0 = "No"; 1 = "Yes" |
| Health Information Technology use: Scheduled a healthcare appointment online | DURING THE PAST 12 MONTHS, have you ever used computers for any of the following …Schedule an appointment with a health care provider | Yes; No | 0 = "No"; 1 = "Yes" |
| Health Information Technology use: Communicated with healthcare provider online | DURING THE PAST 12 MONTHS, have you ever used computers for any of the following …Communicate with a health care provider by email | Yes; No | 0 = "No"; 1 = "Yes" |
| Health Information Technology use: Used internet chat rooms to learn about health topics | DURING THE PAST 12 MONTHS, have you ever used computers for any of the following …Use online chat groups to learn about health topics | Yes; No | 0 = "No"; 1 = "Yes" |
| **HEALTHCARE SYSTEM** | | | |
| Insurance Status | Multiple questions | Uninsured; Private; Medicaid; Medicare; Other | 0 = "Uninsured"; 1 = "Insured" |
| Usual Source of Care | Is there a place that you USUALLY go to when you are sick or need advice about your health? | Yes; There is no place; There is more than one place | 0 = "Usual source of care"; 1 = "No usual source of care" |
| Trouble finding a doctor/provider, past 12m | DURING THE PAST 12 MONTHS, did you have any trouble finding a general doctor or provider who would see you? | Yes; No | 0 = "No"; 1 = "Yes" |
| MD's office not accept you as new patient, past 12m | DURING THE PAST 12 MONTHS, were you told by a doctor’s office or clinic that they would not accept you as a new patient? | Yes; No | 0 = "No"; 1 = "Yes" |
| MD's office not accept your insurance, past 12m | DURING THE PAST 12 MONTHS, were you told by a doctor’s office or clinic that they did not accept your health care coverage? | Yes; No | 0 = "No"; 1 = "Yes" |
| Delayed Medical Care: Couldn't get through on phone | There are many reasons people delay getting medical care. Have you delayed getting care for any of the following reasons in the PAST 12 MONTHS? ..... You couldn't get through on the telephone | Yes; No | 0 = "No"; 1 = "Yes" |
| Delayed Medical Care: Couldn't get appt soon enough | There are many reasons people delay getting medical care. Have you delayed getting care for any of the following reasons in the PAST 12 MONTHS? ..... You couldn't get an appointment soon enough | Yes; No | 0 = "No"; 1 = "Yes" |
| Delayed Medical Care: Wait too long at MD's office | There are many reasons people delay getting medical care. Have you delayed getting care for any of the following reasons in the PAST 12 MONTHS? ..... Once you get there, you have to wait too long to see the doctor | Yes; No | 0 = "No"; 1 = "Yes" |
| Delayed Medical Care: Not open when you could go | There are many reasons people delay getting medical care. Have you delayed getting care for any of the following reasons in the PAST 12 MONTHS? ..... The clinic/doctor's office wasn't open when you could get there | Yes; No | 0 = "No"; 1 = "Yes" |
| Delayed Medical Care: No transportation | There are many reasons people delay getting medical care. Have you delayed getting care for any of the following reasons in the PAST 12 MONTHS? ..... You didn't have transportation | Yes; No | 0 = "No"; 1 = "Yes" |
| Quality of Care (Satisfaction) | In general, how satisfied are you with the healthcare you received in the past 12 months? | Very satisfied; Somewhat satisfied; Somewhat dissatisfied; Very dissatisfied; You haven't had health care in the past 12 months | 0 = "Somewhat/Very Satisfied"; 1 = "Somewhat/Very Dissatisfied or No healthcare in past year" |

**Supplementary Table 2.** Unweighted proportion of the included subjects having each component and sub-item of the social determinants of health (SDOH) score.

| **Components and sub-items of the SDOH score** | **N (%)** |
| --- | --- |
| *Economic stability* |  |
| Never / previously employed | 95 (1.2) |
| No paid sick leave | 3116 (37.8) |
| Low family income | 2050 (24.8) |
| Difficulty paying medical bills | 973 (11.8) |
| Unable to pay medical bills | 462 (5.6) |
| Cost-related medication non-adherence | 672 (8.1) |
| Foregone / delayed medical care due to cost | 680 (8.2) |
| Worried about money for retirement | 2932 (35.5) |
| Worried about medical costs of illness / accident | 2691 (32.6) |
| Worried about maintaining standard of living | 2556 (31.0) |
| Worried about medical costs of normal healthcare | 1732 (21.0) |
| Worried about paying monthly bills | 1714 (20.8) |
| Worried about paying rent / mortgage / housing costs | 1213 (14.7) |
| *Neighborhood, physical environment, and social cohesion* |  |
| Housing was rental / from other arrangement | 1683 (20.4) |
| People in neighborhood did not help each other | 1155 (14.0) |
| There were not people that can be counted on in neighborhood | 988 (12.0) |
| People neighborhood could not be trusted | 904 (11.0) |
| Neighborhood was not close-knit | 2656 (32.2) |
| *Community and social context* |  |
| Psychological distress | 383 (4.6) |
| *Food* |  |
| Food insecurity | 570 (6.9) |
| *Education* |  |
| Could not speak English language well / at all | 127 (1.5) |
| Did not look up health information on internet in the past 12 months | 4157 (50.4) |
| Did not fill a prescription on the internet in the past 12 months | 1104 (13.4) |
| Did not schedule medical appointment on the internet in the past 12 months | 861 (10.4) |
| Did not communicate with healthcare provider by email in the past 12 months | 1315 (15.9) |
| Did not use chat groups to learn about health topics in the past 12 months | 327 (4.0) |
| Less than high school education | 2859 (34.6) |
| *Healthcare system* |  |
| Uninsured | 168 (2.0) |
| No usual source of care | 191 (2.3) |
| Trouble finding a doctor / healthcare provider | 263 (3.2) |
| Not accepted by doctor’s office as new patient | 249 (3.0) |
| Insurance not accepted by doctor’s office | 301 (3.7) |
| Delayed medical care due to not being able to get through on the phone | 242 (2.9) |
| Delayed medical care due to not being able to get an appointment soon enough | 638 (7.7) |
| Delayed medical care due to waiting too long at the doctor’s office | 412 (5.0) |
| Delayed medical care due to the doctor’s office not being open when there was time to visit | 219 (2.7) |
| Delayed medical care due to a lack of transportation | 185 (2.2) |
| Dissatisfied with the quality of care / no healthcare in the past year | 415 (5.0) |

**Supplementary Table 3.** Demographics and components of cardiovascular health in included and excluded subjects. All percentages were unweighted. The non-missing percentages used the sample size of the population without missing data as the denominator, while the missing percentages used the sample size of all excluded subjects as the denominator.

|  | Included | Excluded |
| --- | --- | --- |
| Sample size | 8254 | 8332 |
| Weighted sample size | 10,887,989 | 10,651,338 |
| *Demographics* |  |  |
| Age in years, N (%) |  |  |
| 18-45 | 581 (7.0) | 923 (11.1) |
| 46-64 | 2524 (30.6) | 2771 (33.3) |
| 65 or above | 5149 (62.4) | 4638 (55.4) |
| Male, N (%) | 3755 (45.5) | 3124 (37.5) |
| Race, N (%) |  |  |
| White | 7405 (89.7) | 7217 (86.8) |
| Black / African American | 526 (6.4) | 709 (8.5) |
| American Indian / Alaskan native | 39 (0.5) | 60 (0.7) |
| Asian | 154 (1.9) | 185 (2.2) |
| Multiple race | 130 (1.6) | 142 (1.7) |
| Missing | 0 (0) | 19 (0.2) |
| Sexual orientation, N (%) |  |  |
| Heterosexual | 7995 (96.9) | 7644 (97.0) |
| Missing | 0 (0) | 452 (5.4) |
| *Type of cancer* |  |  |
| Breast, N (%) | 1502 (18.2) | 1613 (19.4) |
| Prostate, N (%) | 1094 (13.3) | 885 (10.6) |
| Lung, N (%) | 274 (3.3) | 288 (3.5) |
| Colorectal, N (%) | 531 (6.4) | 558 (6.7) |
| Skin (melanoma), N (%) | 646 (7.8) | 581 (7.0) |
| Other types, N (%) | 2687 (32.6) | 3097 (37.2) |
| Unknown, N (%) | 2097 (25.4) | 1841 (22.1) |
| *Cardiovascular risk factors* |  |  |
| Hypertension, N (%) | 4883 (59.2) | 4391 (52.8) |
| Missing | 0 (0) | 19 (0.2) |
| Diabetes mellitus, N (%) | 1955 (23.7) | 1680 (20.2) |
| Missing | 0 (0) | 7 (0.1) |
| Hypercholesterolemia, N (%) | 4411 (53.4) | 2873 (34.7) |
| Missing | 0 (0) | 63 (0.8) |
| Smoking, N (%) | 4313 (52.3) | 4163 (50.5) |
| Missing | 0 (0) | 92 (1.1) |
| Physical inactivity, N (%) | 5204 (63.1) | 4745 (66.7) |
| Missing | 0 (0) | 1218 (14.6) |
| Inadequate sleep, N (%) | 1239 (15.0) | 1480 (19.0) |
| Missing | 0 (0) | 534 (6.4) |
| Obesity, N (%) | 2702 (32.7) | 2811 (33.7) |
| Missing | 0 (0) | 0 (0) |
| Excessive alcohol use, N (%) | 458 (8.9) | 427 (9.4) |
| Missing | 3091 (37.5) | 3775 (45.3) |

**Supplementary Table 4.** Proportions of the included and excluded subjects having each component and sub-item of the social determinants of health (SDOH) score. All percentages were unweighted. The non-missing percentages used the sample size of the population without missing data as the denominator, while the missing percentages used the sample size of all excluded subjects as the denominator.

| **Components and sub-items of the SDOH score** | **Included, N (%)** | **Excluded, N (%)** | |
| --- | --- | --- | --- |
|  |  | Present | Missing |
| Sample size | 8254 | 8332 | |
| *Economic stability* |  |  |  |
| Never / previously employed | 95 (1.2) | 169 (2.0) | 5 (0.1) |
| No paid sick leave | 3116 (37.8) | 3212 (36.9) | 787 (9.5) |
| Low family income | 2050 (24.8) | 2655 (36.9) | 1127 (13.5) |
| Difficulty paying medical bills | 973 (11.8) | 1345 (16.2) | 23 (0.3) |
| Unable to pay medical bills | 462 (5.6) | 716 (8.6) | 32 (0.4) |
| Cost-related medication non-adherence | 672 (8.1) | 700 (11.3) | 2156 (25.9) |
| Foregone / delayed medical care due to cost | 680 (8.2) | 991 (11.9) | 0 (0) |
| Worried about money for retirement | 2932 (35.5) | 3273 (41.4) | 432 (5.2) |
| Worried about medical costs of illness / accident | 2691 (32.6) | 3010 (38.1) | 427 (5.1) |
| Worried about maintaining standard of living | 2556 (31.0) | 2977 (37.7) | 431 (5.2) |
| Worried about medical costs of normal healthcare | 1732 (21.0) | 2128 (26.9) | 422 (5.1) |
| Worried about paying monthly bills | 1714 (20.8) | 2240 (28.3) | 420 (5.0) |
| Worried about paying rent / mortgage / housing costs | 1213 (14.7) | 1690 (21.4) | 425 (5.1) |
| *Neighborhood, physical environment, and social cohesion* |  |  |  |
| Housing was rental / from other arrangement | 1683 (20.4) | 2453 (29.5) | 27 (0.3) |
| People in neighborhood did not help each other | 1155 (14.0) | 1307 (17.3) | 785 (9.4) |
| There were not people that can be counted on in neighborhood | 988 (12.0) | 1237 (16.2) | 712 (8.6) |
| People neighborhood could not be trusted | 904 (11.0) | 1148 (15.4) | 869 (10.4) |
| Neighborhood was not close-knit | 2656 (32.2) | 2681 (35.3) | 732 (8.8) |
| *Community and social context* |  |  |  |
| Psychological distress | 383 (4.6) | 433 (5.6) | 561 (6.7) |
| *Food* |  |  |  |
| Food insecurity | 570 (6.9) | 575 (8.1) | 1227 (14.7) |
| *Education* |  |  |  |
| Could not speak English language well / at all | 127 (1.5) | 232 (3.4) | 1457 (17.5) |
| Did not look up health information on internet in the past 12 months | 4157 (50.4) | 3268 (40.4) | 251 (3.0) |
| Did not fill a prescription on the internet in the past 12 months | 1104 (13.4) | 589 (7.3) | 245 (2.9) |
| Did not schedule medical appointment on the internet in the past 12 months | 861 (10.4) | 507 (6.3) | 248 (3.0) |
| Did not communicate with healthcare provider by email in the past 12 months | 1315 (15.9) | 749 (9.3) | 245 (2.9) |
| Did not use chat groups to learn about health topics in the past 12 months | 327 (4.0) | 234 (2.9) | 246 (3.0) |
| Less than high school education | 2859 (34.6) | 3537 (42.7) | 57 (0.7) |
| *Healthcare system* |  |  |  |
| Uninsured | 168 (2.0) | 420 (5.1) | 21 (0.3) |
| No usual source of care | 191 (2.3) | 447 (5.5) | 136 (1.6) |
| Trouble finding a doctor / healthcare provider | 263 (3.2) | 281 (3.4) | 147 (1.8) |
| Not accepted by doctor’s office as new patient | 249 (3.0) | 259 (3.2) | 153 (1.8) |
| Insurance not accepted by doctor’s office | 301 (3.7) | 361 (4.4) | 159 (1.9) |
| Delayed medical care due to not being able to get through on the phone | 242 (2.9) | 226 (2.8) | 159 (1.9) |
| Delayed medical care due to not being able to get an appointment soon enough | 638 (7.7) | 560 (6.9) | 164 (2.0) |
| Delayed medical care due to waiting too long at the doctor’s office | 412 (5.0) | 389 (4.8) | 167 (2.0) |
| Delayed medical care due to the doctor’s office not being open when there was time to visit | 219 (2.7) | 257 (3.2) | 165 (2.0) |
| Delayed medical care due to a lack of transportation | 185 (2.2) | 293 (3.6) | 164 (2.0) |
| Dissatisfied with the quality of care / no healthcare in the past year | 415 (5.0) | 784 (9.8) | 365 (4.4) |

**Supplementary Table 5.** Subgroup analyses for the primary outcome (Cardiovascular Health score). Adjusted risk ratios are displayed with 95% confidence intervals.

|  | N (weighted population) | SDOH score | | | | p_interaction_ |
| --- | --- | --- | --- | --- | --- | --- |
|  |  | Quartile 1 | Quartile 2 | Quartile 3 | Quartile 4 |  |
| *Age* |  |  |  |  |  |  |
| 18-45 | 581 (901913.4) | 1 (reference) | 0.76 [0.55-1.04], p=0.087 | 1.39 [1.04-1.86], p=0.028 | 1.57 [1.22-2.03], p<0.001 | Age 18-45 vs 46-64: 0.69  Age 46-64 vs ≥65: 0.003  Age ≥65 vs 18-45: 0.026 |
| 46-64 | 2524 (3,798,639) | 1 (reference) | 1.06 [0.95-1.17], p=0.30 | 1.14 [1.04-1.25], p=0.006 | 1.37 [1.26-1.49], p<0.001 |  |
| ≥65 | 5149 (6,187,436) | 1 (reference) | 1.04 [1.00-1.08], p=0.070 | 1.12 [1.07-1.17], p<0.001 | 1.19 [1.14-1.25], p<0.001 |  |
| *Sex* |  |  |  |  |  |  |
| Male | 3755 (5,245,061) | 1 (reference) | 1.03 [0.98-1.09], p=0.21 | 1.09 [1.04-1.15], p=0.001 | 1.22 [1.15-1.29], p<0.001 | 0.001 |
| Female | 4499 (5,642,928) | 1 (reference) | 1.04 [0.98-1.11], p=0.18 | 1.18 [1.12-1.25], p<0.001 | 1.38 [1.31-1.46], p<0.001 |  |
| *Race* |  |  |  |  |  |  |
| White | 7405 (9,870,601) | 1 (reference) | 1.04 [1.00-1.08], p=0.075 | 1.15 [1.10-1.20], p<0.001 | 1.32 [1.27-1.38], p<0.001 | 0.051 |
| Non-White | 849 (1,017,388) | 1 (reference) | 0.97 [0.87-1.09], p=0.65 | 1.02 [0.93-1.13], p=0.64 | 1.17 [1.05-1.29], p=0.004 |  |
| *Cancer site* | |  |  |  |  |  |
| Breast | 1502 (1,856,066) | 1 (reference) | 1.04 [0.94-1.15], p=0.48 | 1.15 [1.05-1.27], p=0.002 | 1.39 [1.26-1.52], p<0.001 |  |
| Prostate | 1094 (1,467,371) | 1 (reference) | 1.10 [1.01-1.20], p=0.022 | 1.11 [1.01-1.22], p=0.026 | 1.13 [1.02-1.25], p=0.018 |  |
| Lung | 274 (333,667.2) | 1 (reference) | 1.04 [0.87-1.26], p=0.497 | 1.23 [1.07-1.41], p=0.005 | 1.32 [1.14-1.54], p<0.001 |  |
| Colorectal | 531 (651,411.2) | 1 (reference) | 0.94 [0.81-1.10], p=0.43 | 1.12 [0.98-1.29], p=0.10 | 1.21 [1.07-1.38], p=0.003 |  |
| Skin  (Melanoma) | 646 (881,493.4) | 1 (reference) | 1.03 [0.90-1.18], p=0.66 | 1.15 [1.02-1.30], p=0.026 | 1.33 [1.18-1.51], p<0.001 |  |

**Supplementary Table 6.** Results of sensitivity analyses.

| Outcome | N (weighted population) | SDOH score | | | |
| --- | --- | --- | --- | --- | --- |
|  |  | Quartile 1 | Quartile 2 | Quartile 3 | Quartile 4 |
| *Using ordinal regression instead (OR [95% CI])* | | | | | |
| Primary outcome (CVH) | 8254 (10,887,989) | 1 (reference) | 1.11 [0.97-1.28], p=0.13 | 1.54 [1.34-1.77], p<0.001 | 2.57 [2.21-2.98], p<0.001 |
| *Restricting to those without any known cardiac condition (RR [95% CI])* | | | | | |
| Primary outcome (CVH) | 6011 (8,117,645) | 1 (reference) | 1.06 [1.01-1.11], p=0.028 | 1.17 [1.12-1.23], p<0.001 | 1.34 [1.27-1.40], p<0.001 |
| *Outcome defined as CVH+ excessive alcohol use (RR [95% CI])* | | | | | |
| Alternative outcome (CVH + excessive alcohol use*)* | 5163 (7,074,146) | 1 (reference) | 1.02 [0.97-1.07], p=0.48 | 1.12 [1.06-1.18], p<0.001 | 1.33 [1.27-1.40], p<0.001 |

CI, confidence interval. CVH, Cardiovascular Health. OR, odds ratio. RR, risk ratio. SDOH, social determinants of health.

**Supplementary Table 7.** Results of the exploratory analyses exploring associations between the social determinants of health (SDOH) score and self-reported history of cardiometabolic workup within the past year. Adjusted odds ratios are displayed with 95% confidence intervals.

| Workup | N (weighted population) | SDOH score | | | |
| --- | --- | --- | --- | --- | --- |
|  |  | Quartile 1 | Quartile 2 | Quartile 3 | Quartile 4 |
| Blood pressure | 8250 (10,883,467) | 1 (reference) | 0.57 [0.32-1.03], p=0.063 | 1.34 [0.69-2.57], p=0.38 | 0.89 [0.50-1.58], p=0.70 |
| Fasting glucose | 8141 (10,755,332) | 1 (reference) | 0.91 [0.77-1.08], p=0.29 | 0.98 [0.82-1.17], p=0.80 | 1.07 [0.89-1.28], p=0.47 |
| Cholesterol | 8182 (10,789,312) | 1 (reference) | 0.84 [0.62-1.14], p=0.26 | 0.91 [0.68-1.21], p=0.50 | 0.94 [0.70-1.26], p=0.69 |

**Supplementary Figure 1.** Weighted distribution of the social determinants of health score.


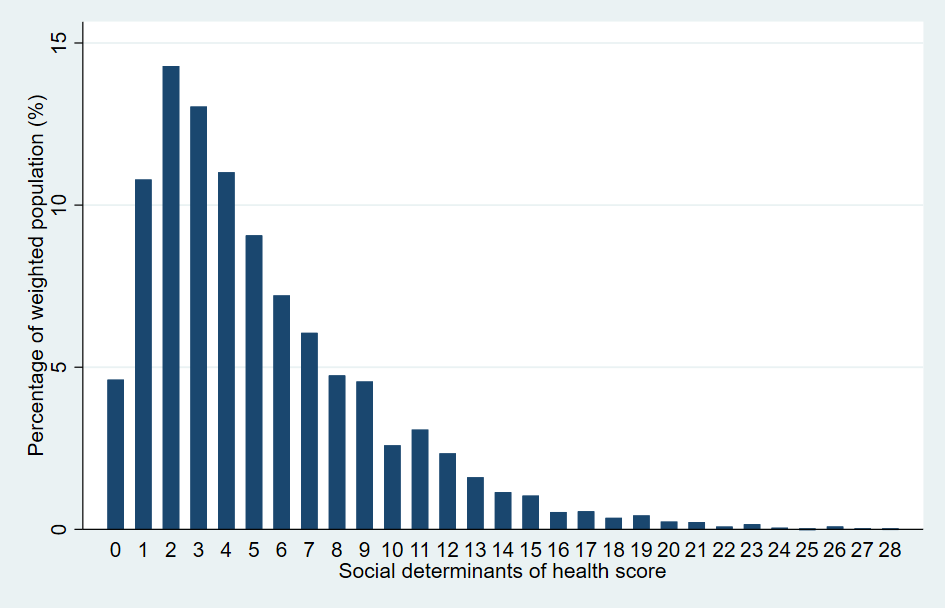


**Supplementary Figure 2.** Weighted distribution of the primary outcome (CVH score).


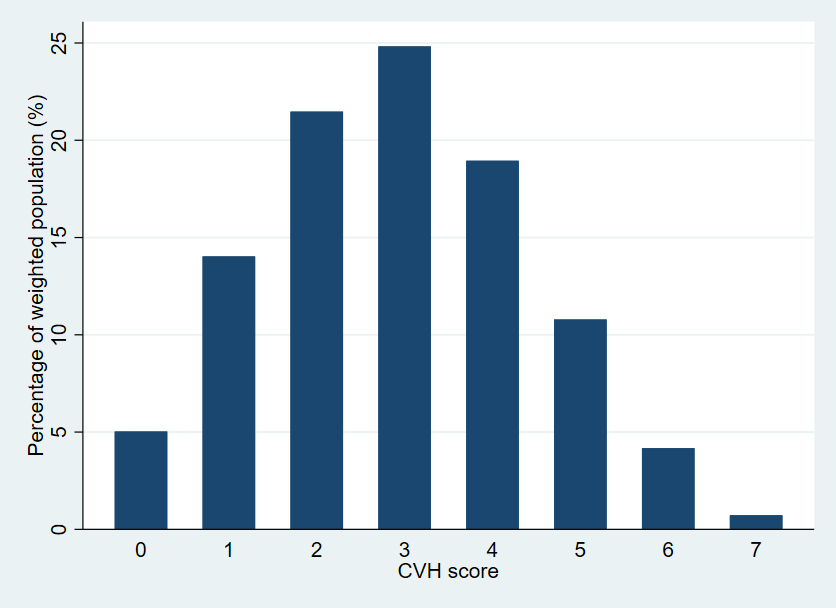

Supplement: Supplemental Material [file mmc1.docx]
